# Plant Nutrient Resource Use Strategies Shape Active Rhizosphere Microbiota Through Root Exudation

**DOI:** 10.3389/fpls.2018.01662

**Published:** 2018-11-23

**Authors:** Julien P. Guyonnet, Martin Guillemet, Audrey Dubost, Laurent Simon, Philippe Ortet, Mohamed Barakat, Thierry Heulin, Wafa Achouak, Feth el Zahar Haichar

**Affiliations:** ^1^Laboratoire d’Ecologie Microbienne, UMR CNRS 5557, Univ Lyon, Université Claude Bernard Lyon 1, UMR INRA 1418, Villeurbanne, France; ^2^Master de Biologie, École Normale Supérieure de Lyon, Université Claude Bernard Lyon 1, Université de Lyon, Lyon, France; ^3^CNRS, UMR 5023 LEHNA, Univ Lyon, Université Claude Bernard Lyon 1, Université Lyon 1, ENTPE, Villeurbanne, France; ^4^CNRS, Laboratory for Microbial Ecology of the Rhizosphere and Extreme Environment, UMR 7265 BIAM, CEA, Aix Marseille Univ, Saint-Paul-lès-Durance, France; ^5^CNRS, FR3098 ECCOREV, Aix Marseille Univ, Aix-en-Provence, France

**Keywords:** active bacterial community, denitrification, microbiota, microbial activities, plant nutrient use strategies, rhizosphere, root exudation, stable isotope probing (SIP)

## Abstract

Plant strategies for soil nutrient uptake have the potential to strongly influence plant–microbiota interactions, due to the competition between plants and microorganisms for soil nutrient acquisition and/or conservation. In the present study, we investigate whether these plant strategies could influence rhizosphere microbial activities *via* root exudation, and contribute to the microbiota diversification of active bacterial communities colonizing the root-adhering soil (RAS) and inhabiting the root tissues. We applied a DNA-based stable isotope probing (DNA-SIP) approach to six grass species distributed along a gradient of plant nutrient resource strategies, from conservative species, characterized by low nitrogen (N) uptake, a long lifespans and low root exudation level, to exploitative species, characterized by high rates of photosynthesis, rapid rates of N uptake and high root exudation level. We analyzed their (i) associated microbiota composition involved in root exudate assimilation and soil organic matter (SOM) degradation by 16S-rRNA-based metabarcoding. (ii) We determine the impact of root exudation level on microbial activities (denitrification and respiration) by gas chromatography. Measurement of microbial activities revealed an increase in denitrification and respiration activities for microbial communities colonizing the RAS of exploitative species. This increase of microbial activities results probably from a higher exudation rate and more diverse metabolites by exploitative plant species. Furthermore, our results demonstrate that plant nutrient resource strategies have a role in shaping active microbiota. We present evidence demonstrating that plant nutrient use strategies shape active microbiota involved in root exudate assimilation and SOM degradation *via* root exudation.

## Introduction

Several studies have shown that plants can host up to 1000 distinct microbial species in their different organs (Bulgarelli et al., 2013; Hacquard et al., 2015). Plant-associated microbiota benefit from their host, since plants provide easily degradable carbon *via* root exudation. Indeed, up to 20% of the carbon fixed by photosynthesis is released in the rhizosphere, which constitutes nutrient sources and signaling molecules for plant–microbe interactions (Bais et al., 2006; Haichar et al., 2014). In turn, plant-associated microbiota influence plant growth by supplying nutrients such as nitrogen (N), phosphorus, or potassium (Singh A. et al., 2004), as well as protecting plants from phytopathogens (Mendes et al., 2011; Samain et al., 2017).

Plants adopt different strategies to interact with their environment for the acquisition or conservation of nutrients (Lambers et al., 2008). These nutrient use strategies could be explained by plant functional traits (McGill et al., 2006; Violle et al., 2007). Indeed, plants with higher photosynthetic capacity and efficient N uptake are referred to as exploitative (or fast-growing) plant species (Gross et al., 2007). By contrast, plants with lower nutrient uptake and lower photosynthetic activities but higher levels of leaf and root dry mass are referred to as conservative (or slow-growing) plant species (Tilman, 1990; Ryser and Lambers, 1995; Aerts and Chapin, 1999). These contrasted strategies of nutrient management influence the input and output of C-resources, from the assimilation of carbon by photosynthesis to its release in the rhizosphere soil *via* root exudation (Guyonnet et al., 2018).

The influence of root exudation of C-resources on microbial communities inhabiting the RAS and colonizing the root systems has been revealed using a stable isotope probing (SIP) approach (Haichar et al., 2008, 2012). These studies displayed significant differences in the active communities in the rhizosphere of four plant species. Although previous studies have explored the link between plant functional traits and plant performance, less is known about the impact of plant nutrient use strategies on bacterial populations that assimilate root exudates, and how they contribute to soil organic matter (SOM) degradation by rhizosphere bacteria.

The aim of this work was to determine the impact of plant nutrient resource strategies on microbial activities of carbon (i.e., respiration) and nitrogen (i.e., denitrification) cycles in soils, as well as on the diversity of active microbiota through root exudation. Specifically, we hypothesized that (i) exploitative plants by exhibiting higher exudation rates than conservative plants will induce an increase in microbial activity (particularly denitrification activity). (ii) Exploitative plants might select a specific bacterial community through root exudation as compared to conservative plants. In addition, by exuding more carbon, exploitative species could promote SOM degradation *via* the priming effect.

To investigate this issue, six plant species in the family *Poaceae* were selected along a nutrient resource gradient, from exploitative to conservative species. The plants were cultivated in the same soil for 10 weeks in a growth chamber and were then submitted to ^13^CO_2_ labeling for 1 week in order to applied SIP. This approach was employed to distinguish the metabolically active bacterial populations that assimilate root exudates from those growing on SOM. In addition, microbial activities (i.e., respiration and denitrification) were measured for each plant rhizosphere.

## Materials and Methods

### Plant Growth

Six perennial C3 grass species distributed along a gradient of plant nutrient use strategies (Gross et al., 2009; Maire et al., 2009; Cantarel et al., 2015; Guyonnet et al., 2018) were examined: two conservative species, *Festuca paniculata* (FP) and *Sesleria caerulea* (SC); and four exploitative species, *Bromus erectus* (BE), *Anthoxanthum odoratum*, (AO), *Dactylis glomerata* (DG), and *Trisetum flavescens* (TF). All studied grasses belong to the *Pooideae* subfamily (Bouchenak-Khelladi et al., 2008). All species were sampled in the field (French Alps) and separated into individual tillers according to Guyonnet et al. (2017). Plants were cultivated on luvisol with no added nitrogen source and were collected at La Côte Saint-André (Isère, France), which is continuously cropped with maize (Guyonnet et al., 2017). The soil was sieved (2 mm mesh size) and 170 g were allocated to each plastic pot. The three individual plants from each species were grown in a greenhouse (13 h day at 22°C/11 h night at 18°C), with an approximate light intensity of 8–10 klux for 11 weeks. Soil moisture was manually controlled. Three pots containing bulk soil (BS) without any plants were incubated as controls under the same conditions.

### Plant ^13^C-Labeling

Continuous labeling was initiated 10 weeks after plant growth, according to a previously reported protocol (Haichar et al., 2008). Plants were placed in a growth chamber (“PHYTOTEC” facility, CEA Cadarache, France) equipped for automatic control of light, temperature, moisture, evapotranspiration, irrigation, and CO_2_ concentration. The day–night period was set at 8 h/16 h, respectively; light intensity was 13.5 klux; maximum daily temperatures ranged from 20 to 22°C; air moisture was adjusted to 80%; and CO_2_ concentration was maintained at 350 μl l^-1^. CO_2_ partial pressure was kept constant by injection of pure ^13^CO_2_ (>99% atom ^13^C purchased from Cortec Net, Paris, France) during active photosynthesis. The isotope excess in the chamber was maintained at >95% atom ^13^C during the 7 days of labeling. Plants and BS were collected after 1 week of ^13^CO_2_ labeling.

### Plant Harvesting

At the end of the labeling experiment, three plants from each population were used to measure microbial activities and to analyze active bacterial diversity. The roots of each plant were manually separated from the root-adhering soil (RAS). Roots were washed carefully with distilled water to remove any remaining soil particles and then immediately frozen in liquid N_2_ and stored at -80°C for DNA extraction. RAS was then carefully separated from the rest of the fine roots and stored at 4°C for microbial activities measurements. Few grams of the RAS were lyophilized, and stored at -80°C for DNA extraction.

### Microbial Activities

Substrate-induced respiration (SIR) and the denitrifying enzymatic assay (DEA) were measured in triplicates according to Bardon et al. (2014) using fresh soil samples (10 g equivalent dry weight of RAS from each plant and BS). SIR was determined as the linear rate of production of CO_2_ during short-term (6 h) incubation under aerobic conditions. Glucose (1.2 mg C g^-1^ dry soil) was added to the soil samples and the soil moisture was brought to 70% water holding capacity. DEA was determined as the linear rate of production of N_2_O during short-term (6 h) incubation under anaerobic conditions. Acetylene gas (C_2_H_2_) was used to inhibit nitrous oxide reductase activity and avoid N_2_ production. Glucose (0.5 mg C g^-1^ dry soil), glutamic acid (0.5 mg C g^-1^ dry soil) and KNO_3_ (50 μg N-NO_3_^-^ g^-1^ dry soil) were added to the soil samples and the soil moisture was brought to 100% water holding capacity. Gasses (CO_2_ and N_2_O) were measured using gas chromatography (Micro GCR3000, SRA Instrument; Marcy-l’Étoile, France).

### DNA Extraction and Gradient Fractionation

DNA was extracted in triplicates from either 3 g of RAS using the “FastDNA^TM^ SPIN Kit for Soil” (MP Biomedicals; United States) or from 0.5 g of fresh roots as described by Ranjard et al. (2003). For extraction from roots, DNA was derived from endophytic microorganisms as well as microorganisms firmly attached to the root surface.

Extracted DNA was quantified using a Qubit^®^ 2.0 Fluorometer (Life Technologies; United States). Extracted DNA from RAS was fractionated by CsCl equilibrium density-centrifugation according to Haichar et al. (2008, 2012), using 4.9 mL “Optiseal Tubes” (Beckman Coulter; United States) in a VTi 65.2 rotor (Beckman Coulter) at 20°C and 45,000 rpm, for 42 h. Buoyant density was obtained by weighing, and DNA was quantified in the CsCl fraction using the Picogreen assay (Molecular Probes). DNA was purified from CsCl salts using the Geneclean turbo kit (Qbiogene; Montreal, QC, Canada).

### Isotope Analysis

The ^13^C content of DNA (total, heavy- and light-DNA fractions) was measured by IRMS (Isoprime 100, Isoprime, Ltd.) coupled with an elemental analyzer (Thermo FlashEA 1112, ThermoElectron) as described in Haichar et al. (2007). 5 μl of DNA was transferred to 3.5-mm × 5-mm “Ultra Clean” tin capsules (Elemental Microanalysis; United Kingdom) and dried for 2 h at 60°C. Tin capsules were then submitted to mass spectrometry.

### Sequencing and Bioinformatics Analysis

Five μl samples (∼50 ng) from each plant’s DNA in triplicates were sent for sequencing to FASTERIS (Switzerland) using the MiSeq illumina technology, comprising fractions representative of light-, heavy-, and root-DNA in triplicates for each plant. The V3–V4 domain of the 16S rRNA gene was amplified with the primers 16S rRNA Fwd primer 3′-CCTACGGGNGGCWGCAG-5′ and 16S rRNA Rev primer 3′-GACTACHVGGGTATCTAATCC-5′. The tagged primers and barcodes were trimmed off by FASTERIS.

We analyzed data using the FROGS software (Escudie et al., 2015). Corrected 16S rRNA gene sequences were clustered into operational taxonomic units (OTUs) using the swarm method (Mahé et al., 2014), in which the distance aggregate was set at *d* = 3. Potential chimeric formations were removed using the VSEARCH tool. OTUs with abundance greater than 0.005% were retained. The taxonomic identification of bacterial and archaeal 16S rRNA genes was performed using the SILVA database release 123 with RDP Classifier (Wang et al., 2007).

#### Network Inference and Graph Display

To study bacteria inhabiting the RAS, OTUs from the RAS found in more than 25% of the samples were selected; the same procedure was performed for root-colonizing bacteria. Microbial networks were constructed using the R package phyloseq (version1.19.1). The pairwise co-occurrence of OTUs over the different samples was assessed using a Bray–Curtis distance (Williams et al., 2014). A proportion table was used instead of an abundance table, so that only the patterns of repartition would be compared and the absolute abundance of any one given OTU would not have an effect. A threshold of 0.12 was used to select significant associations. Every node, and thus every OTU, was positioned on a graph using Cytoscape (version 3.5.1). The horizontal position of every node was calculated according to the preference of the bacteria toward exploitative or conservative plants as follows: $x=\frac{\%\text{exploitative-}\%conservative}{\%\text{exploitative+}\%conservative}$ where %exploitative and %conservative represent the proportion of reads detected in the exploitative and conservative samples, respectively. Additionally, the vertical position of RAS-inhabiting bacteria conveys the inclination toward either the light (L) or heavy (H) compartment: $\frac{\%H-\%L}{\%H+\%L}$ where %H and %L are the proportion of reads detected in the light- and heavy-DNA fractions, respectively.

#### Spider Diagrams

Spider diagrams were plotted in order to present the relative specificity of bacterial phyla toward their hosts. Each community was tested, including bacteria that feed on SOM (light-DNA), bacteria that feed on root exudates (heavy-DNA), and bacteria that colonize root tissues (root-DNA). In each compartment, the total number of OTUs in each phylum was calculated for all the plants and divided by the mean abundance of this particular phylum in all the plants studied. The logarithms of these ratios are displayed.

### Statistical Analyses

For each plant, one-way analysis of variance (ANOVA) and *post hoc* Tukey HSD tests were performed to examine differences in microbial activities between plant strategies. When necessary, data were log- or square-transformed prior to analysis to ensure conformity with the assumptions of normality and homogeneity of variances. When it was not possible (i.e., for exudation variables), non-parametric Kruskal–Wallis and Wilcoxon tests were used. The correlation between microbial activities and root exudation activity was tested by building a linear model with log-transformed microbial activity data. Effects with *p*-value < 0.05 are referred to as significant.

A PCoA (principal coordinates analysis) based on a Bray–Curtis distance was used to compare bacterial diversity retrieved from heavy-, light-, and root-DNA fractions from each plant rhizosphere. For each plant and DNA fraction, the triplicates for OTUs abundance were regrouped. Then, the table of OTU abundance for each fraction from the studied plants and BS samples was treated with the phyloseq package in R, according to McMurdie and Holmes (2013).

The core microbiota were identified using QIIME (Caporaso et al., 2010) and were determined by plotting OTU abundance in the core at 5% intervals (from 50 to 100% of samples). To remove sample heterogeneity, we performed rarefaction on the number of sequences. The approach of rarefaction is to randomly sample the same number of sequences from each sample, and use this data to compare the communities at a given level of sampling effort. We defined the core microbiota of each plant as the OTUs present in 100% of the samples. To determine core microbiota, all samples from each plant were compared across all compartments (light-, heavy-, and root-DNA fractions). Any taxa that were ubiquitous across all samples were then defined as part of the core microbiota of the compartment. Venn diagrams were then constructed from these data, using ‘VennDiagram’ from the R package to show common and unique OTUs within the conservative (FP, SC), the exploitative (AO, BE, DG, TF) and conservative versus exploitative plant groups. All statistical analyses were performed using R 3.1.2 (R Core Team, 2015).

### Data Deposition

The sequence data was deposited at EMBL-ENA public database (http://www.ebi.ac.uk/ena/data/view/PRJEB25083).

## Results

### Impact of Root Exudation on Microbial Activity

**FIGURE 1:**
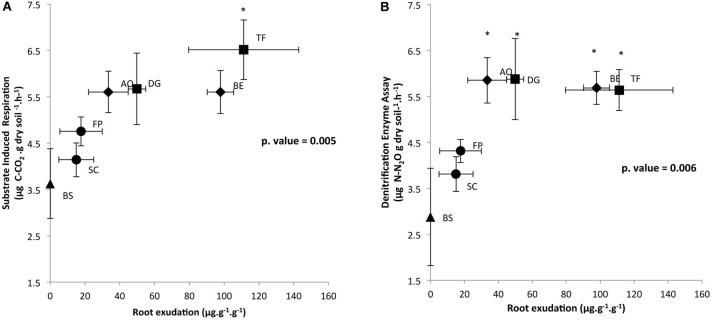
Correlation between **(A)** root exudation rate and SIR and **(B)** root exudation rate and DEA in the rhizosphere of six grasses, distributed on a gradient of plant resource use strategies. Specimens include *Sesleria caerulea* (SC), *Festuca paniculata* (FP), *Anthoxanthum odoratum* (AO), *Berectu erectus* (BE), *Trisetum flavescens* (TF) and *Dactylis glomerata* (DG), as well as BS (control, in the absence of plants). Data represent the means, and error bars represent SE. Significance level is at ^∗^*p* < 0.05 between plants and BS.

In order to determine the impact of root exudation levels of each plant species, measured in our previous work by Guyonnet et al. (2018), on microbial activities, respiratory activities are plotted against root exudation level (Figure 1). Respiratory activities under aerobic and anaerobic conditions that were measured in triplicate on the RAS increased in response to enhanced root exudation activity, as reported in Figure 1. SIR and DEA were log-correlated with root exudation activity (Pearson correlation for DEA and SIR = 0.6; *p* < 0.05). SIR measured on the RAS fraction of SC, FP, AO, BE, and DG plants did not significantly differ from the unique BS sample (Figure 1A). However, SIR was significantly higher for the RAS of TF as compared to SC (Tukey test, *p* < 0.05). Potential denitrification in RAS was significantly higher for AO, BE, DG, and TF than for BS (Tukey test, *p* < 0.05) (Figure 1B). The SIR and DEA activities in the RAS fraction of the four exploitative species (TF, AO, BE, DG) were significantly higher than the RAS fractions of the two conservative species (FP, SC), which did not differ from the BS.

### ^13^C Enrichment of Microbial Communities

To confirm the assimilation of ^13^C-root exudates by soil microbiota, we measured the ^13^C/^12^C ratio of DNA extracted from the RAS recovered from each plant rhizosphere. The δ^13^C of DNA extracted from the RAS of non-labeled plants was approximately -28.7‰ on average, corresponding to natural ^13^C abundance levels. Furthermore, δ^13^C values were, on average, approximately -24.3‰ for BS, -22.2 and -19.2‰ for SC and FP respectively (conservative species), -12.5‰ for BE, 0.01‰ for AO, -5.44‰ for DG, and 13.9‰ for TF (exploitative species).

**FIGURE 2:**
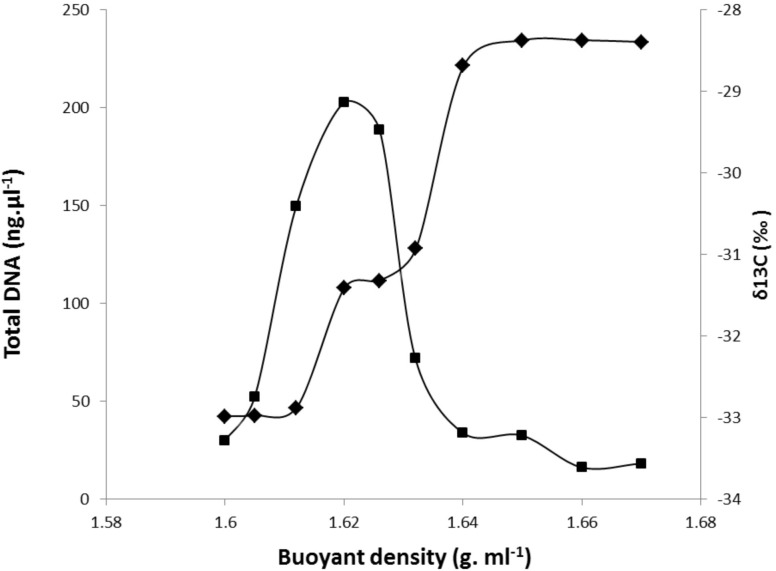
Incorporation of ^13^C-root exudates into microbial community DNA derived from the RAS of *Dactylis glomerata* plants after 1 week of ^13^CO_2_ labeling. ^12^C (light) and ^13^C (heavy) DNA were separated by CsCl density gradient centrifugation. Total DNA was quantified fluorometrically (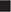), and ^13^C (∂^13^C) values were measured by IRMS within gradient fractions (1–10) (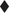).

DNA extracted from each RAS sample was fractionated by ultracentrifugation, and the δ^13^C of the fractions along the gradient were determined. An example of the partitioning of DNA content and ^13^C-labeling among gradient fractions of increasing density for DNA extracted from the RAS of DG plants is presented in Figure 2. The DNA density profile shows a unique peak ranging from 1.62 to 1.63, with ^13^C-labeling measured by IRMS reaching a maximum density between 1.64 and 1.67. Similar profiles were obtained for DNA extracted from the RAS of all other studied grasses. One fraction with a buoyant density of 1.62 g ml^-1^ CsCl was selected as representative of the light-DNA fraction, representing bacterial populations mainly involved in SOM degradation. The fraction with a buoyant density of 1.66 g ml^-1^ CsCl was selected as representative of the heavy-DNA from the bacterial community actively assimilating ^13^C-root exudates (Figure 2).

### Diversity of Active Microbiota Colonizing the Plant Rhizosphere

To investigate the impact of root exudates produced by each plant species on the diversity of active microbiota, three fractions from each plant: the heavy-DNA fraction, the light-DNA fraction, and root-DNA were analyzed by 16S rRNA gene sequencing. Sequencing resulted in 164,640 16S rRNA bacterial sequences corresponding to 1,421 Operational Taxonomic Units per sample, clustered using the swarm method with *d* = 3. The rarefaction curves (rarefaction depth about 2744 sequences), which displayed the observed OTU richness as a function of the sequencing effort, indicate that the sequencing depth was nearly reached to completely comprehend the diversity present in BS, RAS and root tissues of all the plants (Supplementary Figure S1). Alpha-diversity and Inverted Simpson indices were not significantly different between BS and RAS for each plant, although they were significantly decreased in root compartment of the six species (Supplementary Table S1).

To assess structural similarities and/or differences in active microbiota involved in SOM degradation (light-DNA fractions) and root exudate assimilation (heavy-DNA fractions) from the RAS and root tissues (root-DNA fractions) among the plant species, a PCoA plot was generated based on Bray–Curtis distances (Supplementary Figure S2). Bray–Curtis distances provide a measure of community composition differences between samples based on OTU counts, regardless of the taxonomic assignment. Ordinations based on this metric demonstrated a strong separation (Axis 1 = 60.9%) of the bacterial community colonizing plants root tissues (root-DNA fractions) from those inhabiting the RAS involved in SOM degradation (light-DNA fractions) or in root exudate assimilation (heavy-DNA fractions) (Supplementary Figure S2A). The PCoA generated in Supplementary Figure S2B shows a clear distinction between the microbiota retrieved from light-DNA fractions and heavy-DNA fractions from the RAS with those obtained from the BS, indicating the impact of plant *via* root exudates on active microbiota diversity. In addition, by analyzing the diversity of bacteria colonizing the RAS of all the plants, we observed a clear separation and significant differences (*p* < 0.001) between heavy- and light-DNA fractions (triangles vs. circles; Supplementary Figure S2B). However, the influence of the plant species on SOM-degrading bacterial diversity retrieved from light-DNA fractions appears to be marginal (*p* = 0.06) (Figure 2). The PCoA ordination indicates a strong separation between active root-colonizing microbiota (Supplementary Figure S2C, Axis 1 = 50%; Axis 2 = 18.4%) of the six plant species, revealing the high selectivity exerted by root-colonizing microbiota (Supplementary Figure S2C), but without clustering of conservative vs. exploitative species.

**FIGURE 3:**
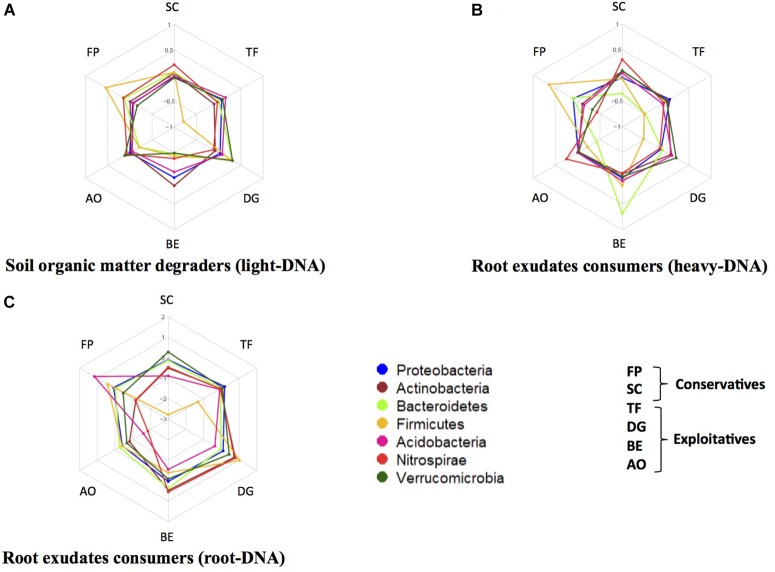
Relative abundance of five major bacterial phyla in the rhizosphere of two conservative plants (*Festuca paniculata* and *Sesleria caerulea*) and four exploitative plants (*Bromus erectus*, *Anthoxanthum odoratum*, *Dactylis glomerata*, and *Trisetum flavescens*). The relative abundances were calculated as the ratio of the abundance of a phylum in a given plant divided by the mean abundance of this phylum in all plants. This was performed for every compartment: **(A)** bacteria feeding on SOM (light-DNA), **(B)** root exudate consumers (heavy-DNA), and **(C)** bacteria colonizing the root tissues.

Several phyla were present in different amounts in the three studied DNA fractions, such as *Proteobacteria*, *Actinobacteria*, *Bacteroidetes*, *Firmicutes*, and *Acidobacteria* (Figure 3). For example, in the light-DNA fraction, the *Firmicutes* phylum was more abundant in the rhizosphere of FP as compared to the other plants, whereas the *Verrucomicrobia* phylum was more abundant in the RAS fraction of DG (Figure 3A). For the heavy-DNA fraction, members of the *Firmicutes* and *Bacteroidetes* phyla were more abundant in FP and BE, respectively, in comparison to the RAS fraction of other plant species (Figure 3B). In addition, the *Nitrospirae* phylum was more abundant in the RAS fraction of AO and SC than in the RAS of other plant species. For root-DNA, the *Acidobacteria* phylum was more abundant on FP roots as compared to other plant species roots (Figure 3C). We also observed that the *Nitrospirae* was more abundant on BE and DG roots, as compared to the other plants. At this level of investigation, two plant species appear to strongly modify the bacterial composition that is influenced directly or indirectly by root exudation. Specifically, the *Firmicutes* are stimulated in the RAS fraction (SOM degraders and root exudate consumers) and the *Acidobacteria* are stimulated in the root tissue fraction of *F. paniculata* (conservative species), whereas the *Bacteroidetes* and *Nitrospirae* are stimulated in the rhizosphere (root exudate consumers only) and the root tissue fraction of *B. erectus* (exploitative species).

### Core Microbiota of Exploitative Plants Species

**FIGURE 4:**
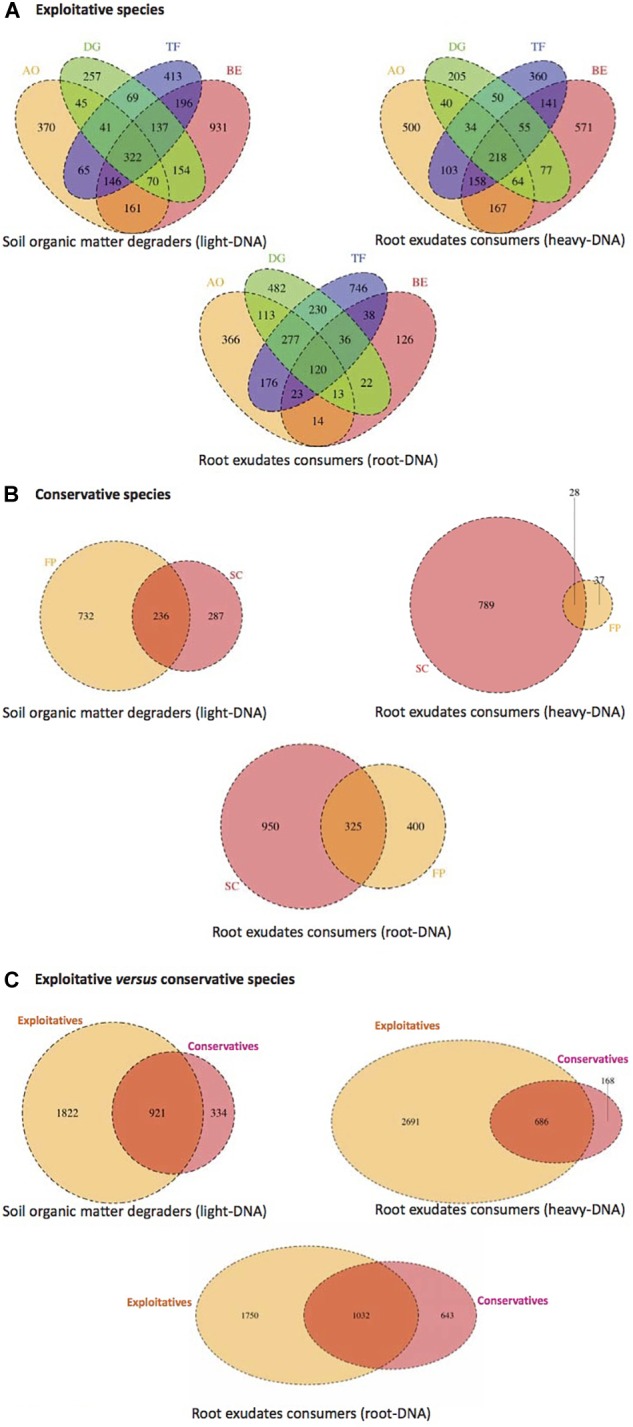
Venn diagram displaying the shared and unique bacterial OTUs at 100% identity among light-DNA (SOM degraders), heavy-DNA, and root-DNA fractions (root exudate consumers) retrieved from **(A)** exploitative species [*Bromus erectus* (BE), *Anthoxanthum odoratum* (AO), *Dactylis glomerata* (DG), and *Trisetum flavescens* (TF)], **(B)** conservative species [*Festuca paniculata* (FP) and *Sesleria caerulea* (SC)], and **(C)** exploitative and conservative species [*Bromus erectus* (BE), *Anthoxanthum odoratum* (AO), *Dactylis glomerata* (DG), *Trisetum flavescens* (TF), *Festuca paniculata* (FP), and *Sesleria caerulea* (SC)].

Operational taxonomic units distribution among all compartments (root tissues, heavy- and light-DNA fractions) were analyzed to highlight the existence of a common core microbiota among the exploitative plants species (Figure 4). As shown in Figure 4A, 322 OTUs were shared by the four exploitative plant species corresponding to the core microbiota involved in SOM degradation. The Venn diagram also reveals unique OTUs corresponding to the microbiota involved in SOM degradation found in the rhizosphere of AO (370), DG (257), TF (413), and BE (931).

In the heavy-DNA fractions corresponding to root exudate consumers in the RAS, 218 OTUs were common to the four exploitative plant species. These shared OTUs can be regarded as the core microbiota involved in root exudate assimilation in the RAS among exploitative plant species. Unique OTUs corresponding to microbiota involved in root exudate assimilation were also detected with high numbers noted in AO (500) and BE (571) plants (Figure 4A). For the bacterial community colonizing the root tissues and involved in root exudate assimilation, 120 common OTUs were identified and could be considered as the root-associated core microbiota. The shared and unique OTUs in the rhizosphere of AO, DG, TF, and BE (light-, heavy-, and root-DNA fractions) are presented in Supplementary Tables S2–S4, respectively.

### Core Microbiota of Conservative Plants Species

As for the exploitative species, the conservative species (SC and FP) shared OTUs corresponding to the core microbiota involved in SOM degradation (236 OTUs), root exudate assimilation in the RAS (28), and in the root tissues (325) (Figure 4B). The Venn diagram also reveals that unique OTUs were observed, with 732 OTUs occupying 76% of the total OTU abundance (968) for SOM degraders in the FP rhizosphere. Furthermore, in the SC rhizosphere, 789 OTUs (96% of all OTUs) were unique to root exudate consumers inhabiting the RAS, and 950 OTUs (75% of all OTUs) were unique to root exudate consumers colonizing the root tissues (Figure 4B). The shared and unique OTUs in the SC and FP rhizosphere (light-, heavy-, and root-DNA fractions) are presented in Supplementary Tables S5–S7, respectively.

### Impact of Plant Nutrient Use Strategies on Diversity of Active Microbiota

The plant nutrient strategy (conservative vs. exploitative) influenced the composition of bacterial communities, as exploitative plants selected more specific SOM degraders (1,822 OTUs), exudate consumers in the RAS (2,691) and root-inhabiting bacteria (1,750) than conservative species (334, 168, and 643 OTUs, respectively) (Figure 4C). However, some bacterial OTUs are common to the conservative and exploitative plant species, including 921 OTUs for the SOM degraders, 686 OTUs for the root exudate consumers in the RAS, and 1,032 OTUs for the root colonizers (Supplementary Tables S8–S10).

### Co-occurrence Networks Among Plant Nutrient Use Strategies

**FIGURE 5:**
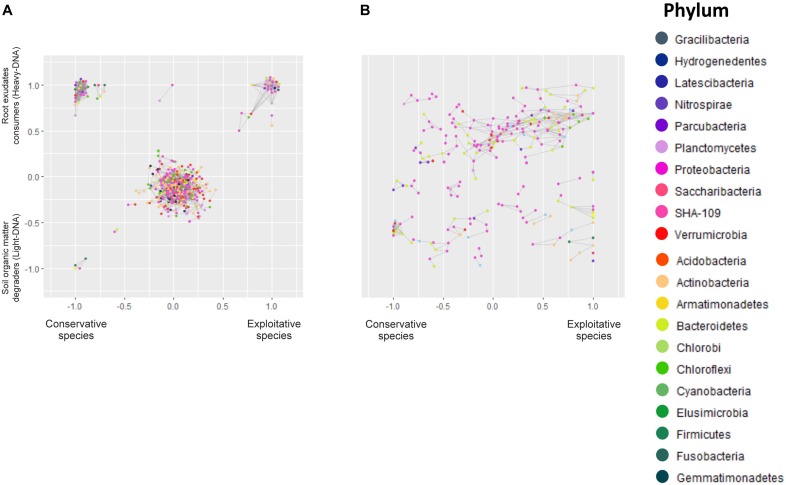
Microbial networks of bacteria inhabiting **(A)** the RAS and involved in root exudate assimilation (heavy-DNA) or SOM degradation (light-DNA). Microbial networks of bacteria colonizing **(B)** the root tissues of conservative plants (*Festuca paniculata* and *Sesleria caerulea*) and exploitative plants (*Bromus erectus*, *Anthoxanthum odoratum*, *Dactylis glomerata*, and *Trisetum flavescens*). Each node represents a gene rRNA sequence, whereas the color indicates the phylum. Bacteria co-occurrence patterns, represented by gray lines, were assessed using a Bray–Curtis distance based on the repartition of OTUs in different plants and compartments. The resulting networks are positioned on a graph representing the gradient from conservative to exploitative plant species on the horizontal axis, and the gradient from SOM degraders (light-DNA) to root exudate consumers (heavy-DNA) on the vertical axis in graph **(A)**. Overlapping nodes were scattered.

Co-occurrence interactions between bacterial taxa were examined in order to determine which taxa respond according to plant strategy (conservative vs. exploitative), carbon nature (fresh carbon/root exudates vs. organic matter) and ecological niche (RAS and root system), thereby providing a means to examine the environmental traits underlying the variation. The microbiota network of the bacterial association is summarized in Figure 5. Overall, the ecological networks were markedly different among exploitative and conservative species.

### Co-occurrence Network Analysis for RAS Microbiota

The resulting microbial network of the OTUs in RAS consisted of 444 nodes (OTUs) linked by 2,555 edges. The average number of neighbors for one node is 11.509, indicating that a given bacterial species will closely share its dispersal pattern with 11.5 other species in the outlined ecological niches (Figure 5). The clustering coefficient represents how the nodes are embedded in their neighborhood, and thus the extent to which they tend to cluster together. This clustering coefficient was 0.35, while the characteristic path length representing the characteristic distance between two random nodes was 3.3. Overall, this indicates that the different niches outlined were dense, with highly connected nodes that clustered with each other.

The structural properties (referred as the number of nodes and connections) of heavy-DNA fraction from the conservative species network were greater than those from exploitative species network, indicating a greater connection and closer relationships between microbial taxa inhabiting the RAS of conservative species (Figure 5A and Supplementary Table S11). Indeed, the number of OTUs belonging to the different phyla in the RAS of conservative species was higher than the exploitative species (i.e., five *Actinobacteria* and five *Bacteroidetes* for conservative species vs. two *Actinobacteria* and two *Bacteroidetes* for exploitative species). Remarkably, *Acidobacteria*, *Chloroflexi* and *Firmicutes* were found exclusively in the microbial network of conservative species, whereas, *Verrucomicrobia* and *Armatimonadetes* were found only in exploitative species microbial network (Supplementary Table S11).

For conservative species, there were strong co-occurrence patterns between members of the classes *Betaproteobacteria*, *Bacilli* and *Fusobacteria*, all involved in SOM degradation. By contrast, co-occurrence for root exudate consumers was observed between members of the classes *Actinobacteria*, *Sphingobacteriia*, *Clostridia*, *Bacilli*, *Alphaproteobacteria*, *Betaproteobacteria*, *Gammaproteobacteria*, *Deltaproteobacteria*, and *Verrucomicrobia* (Supplementary Table S11). For exploitative species, strong co-occurrence was observed between members of the classes *Flavobacteriia*, *Alphaproteobacteria*, *Gammaproteobacteria*, *Betaproteobacteria*, *Actinobacteria* and *Verrucomicrobiae*, all involved in root exudate assimilation. Notably, strong co-occurrence was observed between different classes such as *Alphaproteobacteria* (*Nitrobacter*), *Nitrospira, Actinobacteria*, and *Acidobacteria*. These taxa presented greater versatility, as they were found in the RAS of conservative and exploitative species, and in association with SOM degradation and root exudate assimilation (Supplementary Table S11).

### Co-occurrence Network Analysis for Root-Associated Microbiota

There were 244 nodes and 464 edges in the network representing the root colonizing bacteria. The average number of neighbors for a given node was 3.8, while the clustering coefficient was 0.33 with a characteristic path length of 5.9. The root network is therefore less clustered, and the nodes are less linked to each other.

On the root tissues, regardless of the plant nutrient use strategy, strong co-occurrence patterns were observed between members of the *Proteobacteria*, *Bacteroidetes*, and *Chloroflexi* phyla. However, some co-occurrences were specifically observed in the root tissues of conservative and exploitative species (Figure 5B and Supplementary Table S11). For instance, members of the *Actinobacteria* tended to co-occur exclusively on the roots of exploitative species, whereas members of the *Verrucomicrobia* appeared to be more restricted to the roots of conservative plants (Figure 5B).

## Discussion

In the past decade, numerous studies have documented the importance of plant resource use strategies in community structure and ecosystem functioning (Orwin et al., 2010; Grigulis et al., 2013; Cantarel et al., 2015). However the influence of plant nutrient use strategies through root exudation on microbial activities and diversity remains unexplored. Here, we have investigated how plant nutrient resource strategies influence the active microbiota that inhabit the RAS and colonize the root system through root exudation.

### The Influence of Root Exudation Levels on Microbial Activities Among Plant Nutrient Use Strategies

Root exudates represent the main energy-labile C source for microbiota and are known to affect their structure, activity, abundance and diversity (Grayston et al., 1997; Berg, 2009). In our previous study, we demonstrated that exploitative species exude more carbon than conservative species (Guyonnet et al., 2018), probably due to their larger photosynthetic capacity (Personeni and Loiseau, 2004; De Deyn et al., 2008). Even though root exudation rate of AO and DG plant is somewhat similar to the conservative species, these plants are classified as exploitative species as it present high specific leaf area (SLA) and specific root length (SRL), but low leaf dry mass content (LDMC) and root dry mass content (RDMC) (Guyonnet et al., 2018). Plant nutrient management strategies, which influence root exudate activity (Guyonnet et al., 2018) and quality (Guyonnet et al., 2017), could therefore impact microbial functioning in the RAS. Indeed, SIR and DEA in the RAS were found to increase along with root exudation in the rhizosphere of exploitative plants. Several studies have described an increase in DEA coupled to increased C resources that are released *via* root exudation, e.g., sugars, organic acids, or amino acids (Jones et al., 2004; Mounier et al., 2004; Singh B.K. et al., 2004; Henry et al., 2008). Furthermore, it has been shown that artificial root exudate supply in the soil can increase DEA (Henry et al., 2008). This increase in denitrifying activity can be explained by the fact that denitrification is a heterotrophic activity, and denitrifying microorganisms are able to use root exudates as C-source. In this study, we demonstrated that an increase in DEA is positively correlated with root exudation in RAS, indicating that exploitative grass species characterized by high root exudation activity could stimulate more DEA in the RAS. Rates of DEA and SIR did not differ between exploitative plant species, despite their differences in root exudation activity.

### Influence of Root Exudation on Plant Microbiota

In this study, SIP was applied to root exudates in the rhizosphere of six plant species presenting different nutrient and soil colonization strategies, allowing the differentiation of the active microbiota involved in root exudate assimilation from bacterial communities that degrade SOM.

Differences were observed between light- and heavy-DNA fractions derived from RAS, thereby indicating ^13^C-root exudate assimilation by certain bacterial communities. Indeed, bacterial diversity analysis revealed that the *Sphingobacteria* class is influenced by root exudation in the RAS. *Sphingobacteria* belongs to the *Bacteroidetes* phylum, and these bacteria exhibit copiotrophic attributes (Fierer et al., 2007). These taxa thrive in conditions of elevated C availability (rhizosphere), and display relatively rapid growth rates (Fierer et al., 2012). The genus *Pseudomonas* exhibited significantly increased relative abundance in the heavy-DNA fraction as compared to the light-DNA fraction and root-DNA fractions, confirming that this bacterial genus is very efficient in root exudate assimilation in the RAS.

Unlabeled but reactive populations correspond to organisms probably specialized in the degradation of stable SOM (Fontaine et al., 2003). Furthermore, microorganisms such as *Thermoleophilia* (belonging to the phylum *Actinobacteria*) and certain *Alphaproteobacteria* may produce SOM degrading enzymes, enabling growth at the expense of SOM. *Thermoleophilia* are also known to degrade complex carbon sources in soil (Coombs and Franco, 2003).

The relative abundance of the microbiota inhabiting the root tissues is higher than the abundance of the one inhabiting the RAS. By contrast, the bacterial diversity is lower before the diffusion of nutrients into the RAS, indicating the specific recognition and nutritional selection of microbiota on root tissues. In agreement with several reports (Haichar et al., 2008; El Khalloufi et al., 2016), our data, based on alpha-diversity and inverted-Simpson indices, confirm that the plant selects bacterial populations in the rhizosphere, and more specifically on the root tissues where more intimate plant–bacteria interactions occur. For this reason, the root system is considered as the most selective habitat.

Several bacterial groups that colonize the root tissues, such as *Betaproteobacteria* or *Sphingobacteria* (within the *Bacteroidetes* phylum), are known to be copiotrophic bacteria (Fierer et al., 2012). These bacteria use labile carbon for their metabolism and exhibit a fast growth cycle (r-type strategy). In contrast, some other classes are less abundant in the roots than in the RAS, such as *Actinobacteria*, *Acidobacteria* and *Nistrospira*. These classes, known to be oligotrophic bacteria (Fierer et al., 2007; Koch et al., 2015), are more competitive in different environments with low nutrient concentration and display a low growth cycle (K-type strategy). Remarkably, *Acidobacteria* were overrepresented in the RAS but relatively depleted on root tissues, as previously reported in the rhizosphere of maize (Peiffer et al., 2013) and *Medicago sativa* (El Khalloufi et al., 2016).

*Fibrobacteria* were more abundant on the roots of *A. odoratum* and *S. caerulea* than in any other plants. In addition, this class, which appears to be more abundant in the AO rhizosphere, could be considered as generalist bacteria (Haichar et al., 2008) since it was involved in root exudation assimilation and SOM degradation. Remarkably, *Actinobacteria* colonized the root tissues of all plants in our study, even though they displayed an unequal distribution and were more abundant in the roots of *B. erectus* and *D. glomerata* plants (which presented the largest quantity of carbon exuded into the soil). Although *Actinobacteria* are more frequently located in the rhizosphere soil of many plant species (Barka et al., 2016), some of them are known to form more intimate associations with plants and to colonize their internal tissues, as previously observed for wheat (Coombs and Franco, 2003) and *Arabidopsis thaliana* (Bulgarelli et al., 2013) plants.

### Influence of Plant Nutrient Use Strategies on Active Microbiota

Overall, taxa from the exploitative and conservative plants could be separated into two general categories according to their location: generalists, which are broadly distributed among plants from each plant strategy and represent core microbiota of each fraction (SOM degradation, root exudate assimilation in RAS and on root tissues); and specialists, which are locally abundant in one species and specifically involved in SOM degradation or root exudate assimilation in RAS and on root tissues.

The plants that we examined in this study presented one of two different strategies. Specifically, plants were either conservative species characterized by a slow growth rate, low nutrient uptake capacities and a low root exudate amount, or exploitative species characterized by a fast growth rate and a high root exudate amount (Gross et al., 2007; Guyonnet et al., 2018). Nevertheless, we did observe a core microbiota specialist for SOM degradation and root exudate assimilation in RAS and on root tissues. Our results suggest that these taxa are highly competitive and are able to adapt to the two contrasting plant strategies.

By exuding more carbon into the rhizosphere, exploitative species attract more taxa on root tissues and RAS. They also stimulate more taxa involved in SOM degradation by “priming effect” mechanism (Fontaine et al., 2003). Moreover, Guyonnet et al. (2017) have noted that the composition of primary metabolites is different between exploitative and conservative species. Indeed, exploitative species exude more diverse primary metabolites (i.e., sugar, organic acids), whereas conservative species exude more amino acids. Therefore, in addition to root exudate amount, its composition can be critical in regulating plant microbiota, as also demonstrated by Shade and Handelsman (2011) for wheat and lupin plants.

### Meta-Community Co-occurrence Network

In this study, networks were built not only to determine the links between taxa but also between taxa and plant nutrient use strategies, according to bacterial location (RAS vs. root tissues) and the carbon source (root exudates vs. SOM). Globally, the microbial network of the root is less clustered and connected in comparison to that of the RAS. Although this is related to the smaller number of nodes, it is also linked to the niches that are less delimited than in the RAS.

Some of the observed co-occurrence patterns reveal or confirm interesting ecological patterns for taxa that have not been well-studied. For instance, members of *Verrucomicrobia* tend to co-occur exclusively on the roots of conservative species, suggesting that even though they are abundant and ubiquitous in soils (Sangwan, 2005; Bergmann et al., 2011; Barberán et al., 2012), they may be able to compete for and inhabit more specific and selective niches. In the phylum *Nitrospirae*, *Nitrospira* can co-occur in the RAS of conservative and exploitative species. We observed that *Nitrospira* was associated with SOM degradation and assimilation of root exudates, suggesting that *Nitrospirae* acts as a generalist and is stimulated by plants *via* both strategies. In addition, *Nitrospira*, which can perform complete nitrification (Daims et al., 2015), could provide nitrate to plants, since both the conservative and exploitative species analyzed in previous work present high affinities for nitrate (Grassein et al., 2015).

## Conclusion

Our study demonstrates that plant nutrient use strategies impact microbial activities (respiration and denitrification) *via* root exudation, as well as the diversity of active microbiota involved in root exudate assimilation and SOM degradation. The differences in denitrification activity observed among plant nutrient use strategies could be explained partly by the bacterial diversity, since we detected differences between the plant strategies. These results therefore establish that plants can stimulate microbial activities *via* root exudation, by modifying the diversity of plant microbiota. In addition to bacteria, plants could stimulate other microorganisms in the RAS such as fungi that play a key role in denitrification process (Maeda et al., 2015) and SOM degradation (Buée et al., 2009), and which could also be affected by plant nutrient management strategy.

## Author Contributions

FZH designed the experiments. JG conducted the experiments. LS performed the ^13^C measurement. JG, AD, MG, MB, PO, WA, TH, and FZH analyzed the results. FZH, JG, WA, and TH wrote the manuscript. All authors have read and approved the final manuscript.

## Conflict of Interest Statement

The authors declare that the research was conducted in the absence of any commercial or financial relationships that could be construed as a potential conflict of interest.

## Supplementary Material

Figure S1 Bacterial diversity in the rhizosphere of *Bromus erectus* (BE), *Anthoxanthum odoratum* (AO), *Dactylis glomerata* (DG), *Trisetum flavescens* (TF), *Festuca paniculata* (FP) and *Sesleria caerulea* (SC), as characterized by rarefaction curves of OTUs clustered using the swarm method with *d* = 3. H: heavy-DNA fraction; L: light-DNA fraction; R: root-DNA.

Figure S2 Principal coordinate analysis (PCoA) profile of the active bacterial diversity (using Bray–Curtis distances) colonizing the RAS and inhabiting the root tissues of *Bromus erectus* (BE), *Anthoxanthum odoratum* (AO), *Dactylis glomerata* (DG), *Trisetum flavescens* (TF), *Festuca paniculata* (FP), and *Sesleria caerulea* (SC). **(A)** PCoA of active microbiota obtained from light- and heavy-DNA fractions from the BS, and from the RAS and root-DNA fractions. **(B)** PCoA of active microbiota involved in SOM degradation (light-DNA fractions) and in root exudate consumption (heavy-DNA fractions). **(C)** PCoA of active microbiota colonizing the root tissues.

Table S1 Sample coverage, species richness and species diversity indices.

Table S2 Taxonomic affiliation of OTUs retrieved from light-DNA fractions (SOM degraders): (file 1) shared by exploitative plants (DG, TF, BE, and AO) and representing the core microbiota; (file 2) present in AO only; (file 3) present in BE only; (file 4) present in DG only; (file 5) present in TF only.

Table S3 Taxonomic affiliation of OTUs retrieved from heavy-DNA fractions (root exudate consumers): (file 1) shared by exploitative plants (DG, TF, BE, and AO) and representing the core microbiota; (file 2) present in AO only; (file 3) present in BE only; (file 4) present in DG only; (file 5) present in TF only.

Table S4 Taxonomic affiliation of OTUs retrieved from root-DNA fractions (root exudate consumers): (file 1) shared by exploitative plants (DG, TF, BE, and AO) and representing the core microbiota; (file 2) present in AO only; (file 3) present in BE only; (file 4) present in DG only; (file 5) present in TF only.

Table S5 Taxonomic affiliation of OTUs retrieved from light-DNA fractions (SOM degraders): (file 1) shared by conservative plants (FP and SC) and representing the core microbiota; (file 2) present in FP only; (file 3) present in SC only.

Table S6 Taxonomic affiliation of OTUs retrieved from heavy-DNA fractions (root exudate consumers): (file 1) shared by conservative plants (FP and SC) and representing the core microbiota; (file 2) present in FP only; (file 3) present in SC only.

Table S7 Taxonomic affiliation of OTUs retrieved from root-DNA fractions (root exudate consumers): (file 1) shared by conservative plants (FP and SC) and representing the core microbiota; (file 2) present in FP only; (file 3) present in SC only.

Table S8 Taxonomic affiliation of OTUs retrieved from light-DNA fractions (SOM degraders): (file 1) shared by exploitative (DG, BE, AO, and TF) and conservative plants (FP and SC) and representing the core microbiota; (file 2) present in exploitative plants only; (file 3) present in conservative plants only.

Table S9 Taxonomic affiliation of OTUs retrieved from heavy-DNA fractions (root exudate consumers): (file 1) shared by exploitative (DG, BE, AO and TF) and conservative plants (FP and SC) and representing the core microbiota; (file 2) present in exploitative plants only; (file 3) present in conservative plants only.

Table S10 Taxonomic affiliation of OTUs retrieved from root-DNA fractions (root exudate consumers): (file 1) shared by exploitative (DG, BE, AO and TF) and conservative plants (FP and SC) and representing the core microbiota; (file 2) present in exploitative plants only; (file 3) present in conservative plants only.

Table S11 Table of the different bacteria present in the co-occurrence analysis (Figure 5). Each sheet displays the taxonomic affiliation of the bacterial communities uncovered for the RAS, including root exudate consumers of exploitative plant species (Exploit_heavy DNA fractions), root exudate consumers of conservative plant species (Cons_heavy DNA fractions), SOM degraders from the rhizosphere of conservative species (Cons_light DNA fractions), and the bacteria common to both strategies. The same information was prepared for the bacteria found in the root network (Figure 5), and the position on the gradient from conservative to exploitative was reported.
