# Supplementary figures and images for: Plant Nutrient Resource Use Strategies Shape Active Rhizosphere Microbiota Through Root Exudation
Source: Front Plant Sci. 2018 Nov 23;9:1662. doi: 10.3389/fpls.2018.01662 (PMC6265440; doi:10.3389/fpls.2018.01662)

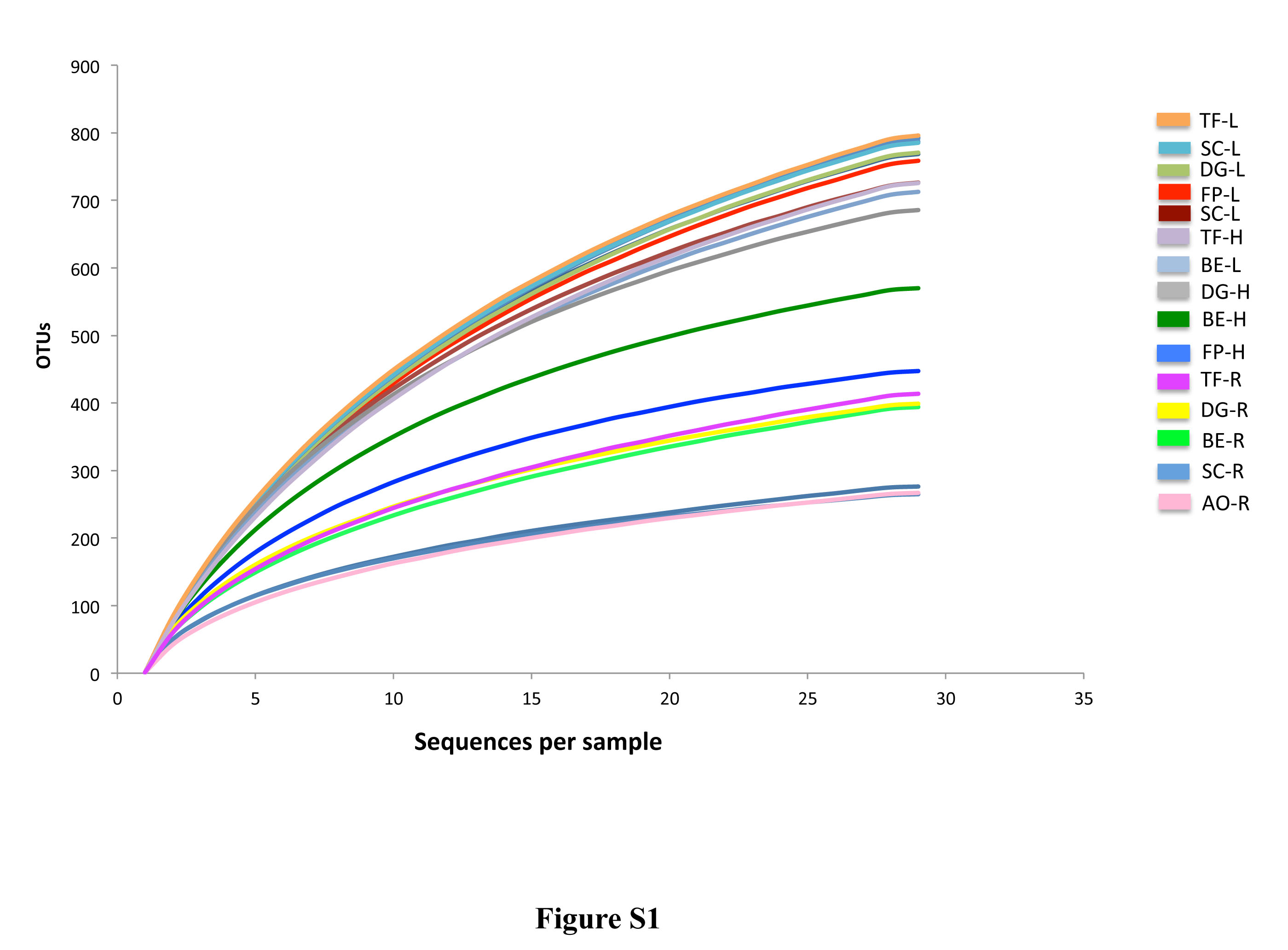

Supplement: Figure S1 — Bacterial diversity in the rhizosphere of Bromus erectus (BE), Anthoxanthum odoratum (AO), Dactylis glomerata (DG), Trisetum flavescens (TF), Festuca paniculata (FP) and Sesleria caerulea (SC), as characterized by rarefaction curves of OTUs clustered using the swarm method with d = 3. H: heavy-DNA fraction; L: light-DNA fraction; R: root-DNA. [file Image_1.JPEG]

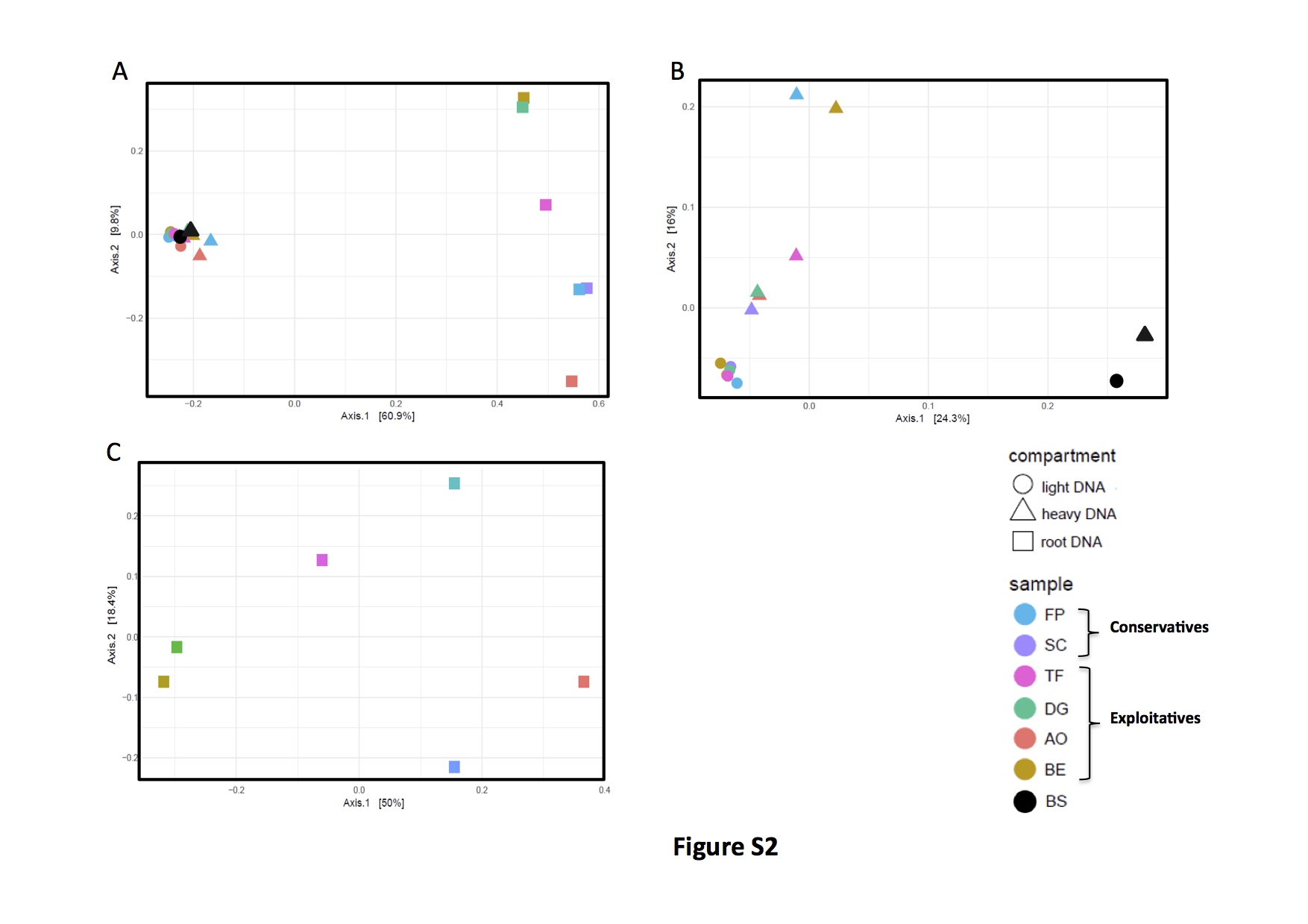

Supplement: Figure S2 — Principal coordinate analysis (PCoA) profile of the active bacterial diversity (using Bray–Curtis distances) colonizing the RAS and inhabiting the root tissues of Bromus erectus (BE), Anthoxanthum odoratum (AO), Dactylis glomerata (DG), Trisetum flavescens (TF), Festuca paniculata (FP), and Sesleria caerulea (SC). (A) PCoA of active microbiota obtained from light- and heavy-DNA fractions from the BS, and from the RAS and root-DNA fractions. (B) PCoA of active microbiota involved in SOM degradation (light-DNA fractions) and in root exudate consumption (heavy-DNA fractions). (C) PCoA of active microbiota colonizing the root tissues. [file Image_2.JPEG]
