# Supplementary material for: Plant Nutrient Resource Use Strategies Shape Active Rhizosphere Microbiota Through Root Exudation
Source: Front Plant Sci. 2018 Nov 23;9:1662. doi: 10.3389/fpls.2018.01662 (PMC6265440; doi:10.3389/fpls.2018.01662)
Supplement: Table S1 — Sample coverage, species richness and species diversity indices. [file Table_1.pdf]

Table S1.

Compartment	Alpha		Inverse Simpson	
	Mean	SE	Mean	SE
**SC_light**	314.67	10.66	148.88	14.43
**SC_heavy**	297.33	6.22	122.06	39.77
**SC_roots**	128.67	12.43	22.42	3.28
**FP_light**	293.33	4.82	118.78	3.60
**FP_heavy**	211.67	102.40	97.45	53.79
**FP_roots**	125.33	35.28	24.68	6.69
**AO_light**	301.67	10.00	134.79	10.95
**AO_heavy**	299.33	6.90	131.77	24.74
**AO_roots**	118.33	10.07	8.64	0.79
**BE_light**	291.67	16.64	118.97	27.92
**BE_heavy**	257.00	72.43	101.47	58.71
**BE_roots**	175.00	16.94	38.74	18.95
**DG_light**	316.33	8.56	144.32	10.30
**DG_heavy**	295.67	18.01	151.50	13.78
**DG_roots**	182.33	17.26	57.13	19.82
**TF_light**	309.00	5.00	132.98	11.77
**TF_heavy**	283.67	61.96	114.30	76.06
**TF_roots**	174.67	21.92	42.07	16.96
**BS_light**	306.33	5.45	147.79	12.10
**BS_heavy**	297.33	4.03	121.31	14.53
